# Outcomes and indications for emergency thoracotomy after adoption of a more liberal policy in a western European level 1 trauma centre: 8-year experience

**DOI:** 10.1007/s13304-018-0607-4

**Published:** 2018-12-26

**Authors:** Edoardo Segalini, Luca Di Donato, Arianna Birindelli, Alice Piccinini, Alberto Casati, Carlo Coniglio, Salomone Di Saverio, Gregorio Tugnoli, Bologna Trauma Team collaborative group, Giovanni Gordini, Andrea Biscardi, Sergio Nicola Forti Parri, Barbara Bonfanti, Kenji Kawamukai, Nicola Lacava, Cristian Lupi, Piergiorgio Cavalli, Enrico Ferri, Aimone Giugni, Sara Trentini, Marzia Baldazzi, Silvia Villani, Jessica McKee, Antonio Affinita, Elena Mengozzi, Nicola Montanari, Mauro Podda, Sherman Kwan

**Affiliations:** 1 Trauma Surgery Unit, Maggiore Hospital Regional Emergency Surgery and Trauma Center, Bologna Local Health District, Bologna, Italy; 2 General and Emergency Surgery, Arcispedale S. Maria Nuova Hospital, Reggio Emilia, Italy; 3 Division of Trauma and Surgical Critical Care, LAC + USC Medical Center, University of Southern California, Los Angeles, CA USA; 4 Trauma Intensive Care Unit, Maggiore Hospital Regional Emergency Surgery and Trauma Center, Bologna Local Health District, Bologna, Italy; 5 Cambridge Colorectal Unit, Cambridge University Hospitals NHS Foundation Trust, Addenbrooke’s Hospital, Cambridge Biomedical Campus, Hills Road, Cambridge, CB2 0QQ UK

**Keywords:** Emergency resuscitative thoracotomy, Emergency department thoracotomy, Emergency thoracotomy, Clamshell thoracotomy, Blunt trauma, Penetrating trauma, Cardiac repair, Aortic cross-clamping, Open cardiac massage

## Abstract

The role of emergency thoracotomy (ET) in blunt trauma is still a matter of debate and in Europe only a small number of studies have been published. We report our experience about ET both in penetrating and blunt trauma, discussing indications, outcomes and proposing an algorithm for patient selection. We retrospectively analysed patients who underwent ET at Maggiore Hospital Trauma Center over two periods: from January 1st, 2010 to December 31st, 2012, and from January 1st, 2013 to May 31st, 2017. Demographic and clinical data, mechanism of injury, Injury Severity Score, site of injury, time of witnessed cardiac arrest, presence/absence of signs of life, length of stay were considered, as well as survival rate and neurological outcome. 27 ETs were performed: 21 after blunt trauma and 6 after penetrating trauma. Motor vehicle accident was the main mechanism of injury, followed by fall from height. The mean age was 40.5 years and the median Injury Severity Score was of 40. The most frequent injury was cardiac tamponade. The overall survival rate was 10% during the first period and 23.5% during the second period, after the adoption of a more liberal policy. No long-term neurological sequelae were reported. The outcomes of ET in trauma patient, either after penetrating or blunt trauma, are poor but not negligible. To date, only small series of ET from European trauma centres have been published, although larger series are available from USA and South Africa. However, in selected patients, all efforts must be made for the patient’s survival; the possibility of organ donation should be taken into consideration as well.

**Electronic supplementary material:**

The online version of this article (10.1007/s13304-018-0607-4) contains supplementary material, which is available to authorized users.

## Introduction

Emergency thoracotomy (ET) is a potentially life-saving procedure with outcomes being the most favourable in penetrating trauma [1, 2], when pre-hospital CPR does not exceed 15 min [3], asystole is the presenting rhythm without pericardial tamponade [3] and the America College of Surgeons Committee on Trauma (ACS-COT) practice guidelines are followed [4]. However, this is based in North America where penetrating injury is the predominant mechanism of injury (MOI) and the trauma systems are equipped and trained to conduct ET [1]. When examining the European experience with ET, Narvestad [5] disseminates that European centres see a fraction of the penetrating trauma seen in North America (< 10% penetrating trauma) and ET is rarely indicated in blunt trauma patients because of reported dismal outcomes [6]. However, within the eight European ET studies examined by Narvestad [5], survival rates ranged from 0 to 59.1%, with two studies [7, 8] demonstrating an almost equal ratio of blunt to penetrating injury survival.

**Fig. 1:**
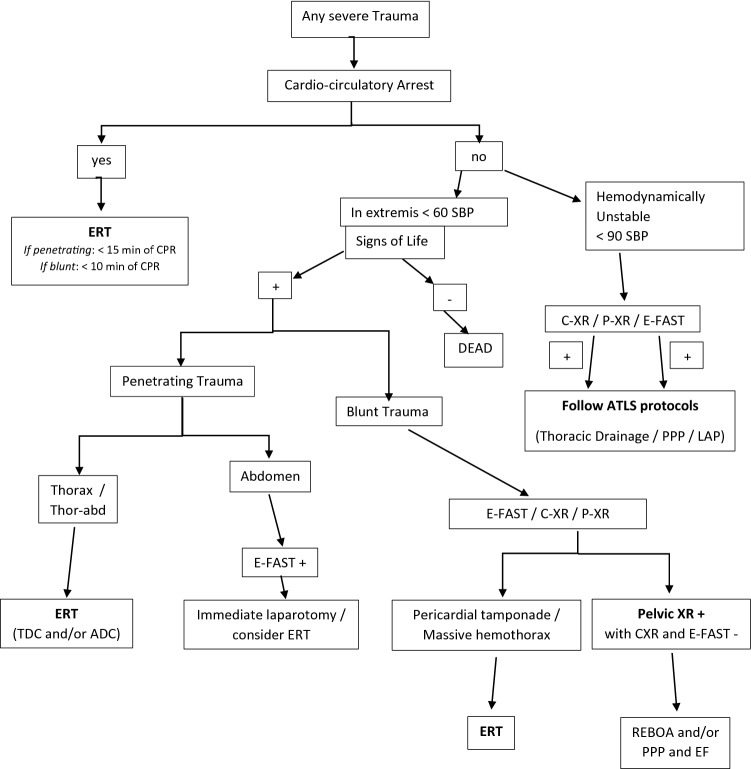
Flowchart with decision-making pathway for emergency thoracotomy (ET) in Maggiore Hospital Trauma Center of Bologna. CPR: cardio-pulmonary resuscitation. SBP: systolic blood pressure. *ATLS* advanced trauma life support, *SoL* signs of life (cardiac electrical activity, motor or respiratory effort, or pupillary activity), *TDC* Thoracic Damage Control, *ADC* Abdominal Damage Control, *CXR* chest X-ray, *PXR* pelvic X-ray, *E-FAST* extended focused assessment with sonography for trauma, *REBOA* resuscitative endovascular balloon occlusion of the aorta, *EF* external fixation

**Fig. 2:**
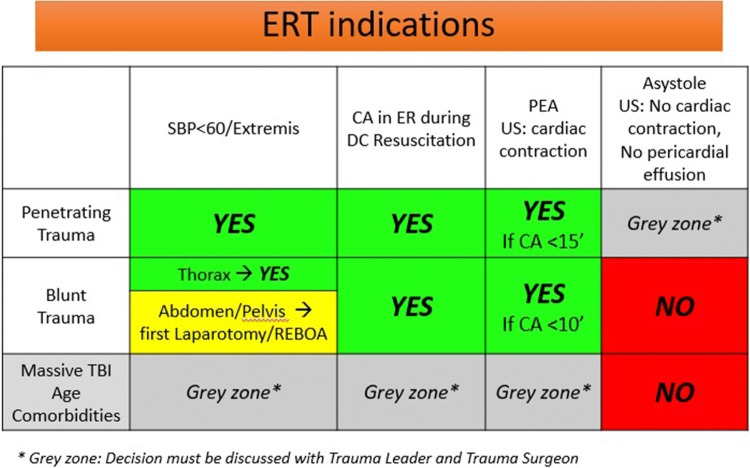
This table is daily used in the ER and it is more user friendly in the critical situations, such as during the survey of a major trauma patient. *SBP* systolic blood pressure, *CA* cardiac arrest, *ER* emergency room, *DC* damage control, *PEA* pulseless electrical activity, *US* ultrasonography, *TBI* traumatic brain injury

Regardless of the MOI in which ET is performed, it is an intervention that carries significant morbidity and mortality which combined with health care reform [3] has led to considerable scrutiny. [6, 9–12]. The Bologna Maggiore Hospital is one of the three hub hospitals of the Regional Trauma System of Emilia-Romagna, Italy. From 1989 to 1994, 20 ETs were performed for blunt trauma with a mortality rate of 100%. In consideration of these results, ET was abandoned. However, in the past two decades, European-based ET survival rates including those performed in blunt trauma have been published which has generated renewed interest in this procedure. In 2010, considering these reports [1], the Trauma Team of Maggiore Hospital changed the policy and decided to reimplement ET, according to international guidelines [10]. This study aims to review and critically analyse data surrounding ETs performed at the Bologna Maggiore Hospital, performed over two different periods: between January 1st, 2010 and December 31st, 2012, and between January 1st, 2013 and May 31st, 2017. In addition, we aimed to use these data to develop a clinical algorithm for ET within the European context (Figs. 1, 2). Regarding the patients with systolic blood pressure between 60 and 90 mmHg, the indication to perform ET depends on the results of the primary survey, in accordance with the Advanced Trauma Life Support algorithm, because these patients are not in extremis and, thus, it is possible to perform the basic diagnostic procedure, such as chest X-ray, pelvis X-ray and E-FAST, in the Emergency Room to identify life-threatening conditions. The policy of ET has been changed further since 2013 with a more liberal policy enlarging the indications for ET, along with a specific better training achieved by a dedicated trauma team and a standardized surgical technique of ET (including always a Clamshell incision followed by better exposure and easier resuscitative manoeuvres on the heart/mediastinum/lung/thoracic aorta) proposed by the trauma team.

## Materials and methods

This was a retrospective chart review of all patients aged 18 years or more, that underwent ET at the Bologna Maggiore Hospital Trauma Center during two separate time periods: January 1st, 2010–December 31st, 2012, and January 1st, 2013–May 31st, 2017. The decision to perform ET during the study period was made by the trauma anaesthetist and the attending trauma surgeon.

ET is a potentially life-saving procedure in selected patients; the correct selection of the patients has a key role and it is still being debated, especially in blunt trauma where the outcome is believed to be poor. The main purposes of an ET are (a) release a pericardial tamponade and control a cardiac haemorrhage (supplementary figure 3; (b) control other sources of intrathoracic bleeding, such as great vessels or pulmonary lacerations; (c) perform open cardiac massage and internal defibrillation; (d) evacuate massive air embolism and clamp or twist the pulmonary hilum (supplementary figure 4) (video 1); (e) perform thoracic aorta cross-clamping, achieving proximal control of the descending thoracic aorta, during major intra-abdominal bleeding [10] (video 2).

The ET procedures were performed by the trauma surgeon on call. A clamshell thoracotomy is commonly performed (supplementary figure 5) as it provides better exposition of the thoracic organs and vessels [9], rather than a simple antero-lateral left-sided thoracotomy (supplementary figure 6). [13].

We considered ET performed both in emergency room and in operating room; in fact, according to Hunt P.A. [20], ET should be defined as a procedure occurring either in emergency department or in the operating room, as an integral part of the initial resuscitation. Thus, we concur with this definition and consider OR thoracotomies as ET as this can be considered part of the initial resuscitative manoeuvres.

The ET was considered successful when the patient survived the procedure and we classified these patients as “early survivors”. The overall survival rate was calculated based on those that survived after 30 days.

Demographic and clinical data were collected such as MOI, Injury Severity Score (ISS), the location of injury, the time of witnessed cardiac arrest, signs of life (SoL), length of stay, survival rate and long-term outcome in terms of neurological sequelae. Neurological outcome was assessed by a Neurologist. Statistical software (SPSS ver.13.0) has been used for statistical analyses. Statistical significance has been tested with Chi-square test or Fisher exact test as appropriate. The work has been reported in line with the STROCSS criteria [14]. The study was approved by the Institutional Ethical Board of the Maggiore Hospital.

## Results

Over the study period, 27 patients who received ET were identified, males accounted for 74% (*n* = 20) and females 26% (*n* = 7). The mean age was 40.5 years (range 25–53). Patients present median ISS of 40 (IQR 13). There were 21 (77.8%) ETs for blunt trauma and six ETs for penetrating injuries (22.2%). The most common MOI for blunt trauma was motor vehicle collision (MVC) (56%) and falls from height (20%), followed by motorbike collision (12%). Stabbing accounted for 66% of all penetrating injuries, followed by gunshot wound (33%). Eight patients (29.7%) arrived at the emergency department (ED) after a witnessed cardiac arrest with cardio-pulmonary resuscitation (CPR) started on site or during transport, less than 10 min before admission. Thirteen patients (48.1%) were admitted in extremis (systolic blood pressure (SBP) < 60 mmHg) and 6 (22.2%) patients were considered hemodynamically unstable (SBP 60–90 mmHg). 13 (48.1%) ETs were performed in ED and 14 (51.9%) in the trauma operating room (OR) for those patients with profound and refractory shock but not yet in cardiac arrest.

During the first period, only one patient (10%) survived 30 days post-ET for penetrating injury whilst there were four “early survivors” (40%): two after blunt trauma and two after penetrating (Table 1). After the adoption of a more liberal policy for ET in 2013, four patients (23.5%) survived ET, one (5.8%) after blunt thoracic injury and 3 (17.6%) after penetrating trauma. During this second period, there were eight (47%) “early survivors”: four after penetrating trauma and four after blunt trauma (Table 2).

**Table 1:** The outcomes after ET during the period between January 1st, 2010 and December 31st, 2012, before the adoption of the more liberal policy

2010–2012	Total	Dead during ERT	Survived to the ERT	Dead in ICU	Survived
Patients	10	6 60%	4 40%	3 30%	1 10%
Blunt trauma	8 80%	6 60%	2 20%	2 20%	0
Penetrating trauma	2 20%	0	2 20%	1 10%	1 10%
Cardiac arrest	3 30%	3 30%	0	0	0
In extremis	5 50%	3 30%	2 20%	2 20%	0
Unstable	2 20%	0	2 20%	1 10%	1 10%

**Table 2:** The outcomes after the adoption of the more liberal policy, during the period between January 1st, 2013 and May 31st, 2017

2013–2017	Total	Dead during ERT	Survived to the ERT	Dead in ICU	Survived
Patients	17	9 52.9%	8 47%	4 23.5%	4 23.5%
Blunt trauma	13 76.5%	9 52.9%	4 23.5%	3 17.6%	1 5.8%
Penetrating trauma	4 23.5%	0	4 23.5%	1 5.8%	3 17.6%
Cardiac arrest	5 29.4%	3 17.6%	2 11.7%	1 5.8%	1 5.8%
In extremis	8 47%	4 23.5%	4 23.5%	2 11.7%	2 11.7%
Unstable	4 23.5%	2 11.7%	2 11.7%	1 5.8%	1 5.8%

Among the survivors during the period between 2013 and 2017, one patient presented in cardiac arrest following penetrating trauma, two were in extremis hemodynamically (one blunt trauma and one penetrating) and one suffered from severe hemodynamic instability after penetrating trauma. In these patients, cardiac tamponade was the most frequent injury detected (2 patients), followed by severe pulmonary laceration (1 patient). Pericardiotomy and internal cardiac massage were the main manoeuvres performed. Neurological long-term outcome was favourable in all survivors, without neurological impairment or other impediments to independence in activities of daily living and it was assessed by a neurologist with a complete neurological clinical assessment. The average length of hospital stay among the survivors was 35 days.

## Discussion

Various ET guidelines have been published including suggested algorithms to select and manage these patients. Beside the injury mechanism, algorithms consider the presence or absence of signs of life (SoL) and vital signs [10]. Often these terms are used synonymously; however, SoL indicate cardiac electrical activity, motor or respiratory effort, or pupillary activity. In contrast, vital signs refer to palpable pulses, blood pressure or spontaneous breath.

The first ET guidelines were published in 2001 by the American College of Surgeon Committee on Trauma and suggested ET for penetrating trauma with witnessed SoL, on scene followed by a rapid transport, whilst ET in blunt trauma only if SoL were initially present but then lost at the trauma centre [1]. In 2012, the Western Trauma Association (WTA) produced a paper, which clarified indications for ET, considering two key factors: injury mechanism and duration of cardio-pulmonary resuscitation (CPR). The WTA suggested ET if the time to initiation of CPR was within 10 min of blunt trauma and within 15 min after a penetrating trauma [2]. Three years later, in 2015, the Eastern Association for the Surgery of Trauma (EAST) performed a systematic review of 72 studies about ET, including more than 10,000 ETs. EAST published evidence-based guidelines that strongly recommended ET after penetrating thoracic injury in patients that arrived in ED pulseless but with SoL. According to these guidelines, ET was conditionally recommended for patients that arrived to ED pulseless and without SoL after a penetrating thoracic injury, and for patients presenting pulseless with a penetrating extra-thoracic injury, with or without SoL. These guidelines also proposed a conditional recommendation for ET in pulseless patients with SoL, after a blunt trauma; however, it also recommended that patients with blunt trauma arriving in ED without SoL, ET should not be performed [11].

From 1960s, many papers on ET have been published, reflecting the level of international experience and discussion on this debated procedure. In 1966, Beall and colleagues [12] published a paper about the management of heart trauma, explaining that emergency thoracotomy could be a resuscitative procedure when performed for penetrating, life-threatening, chest injuries [12]. During the following years, the literature reports many papers in favour of ET in case of penetrating trauma, with good survival rates when performed in selected patients [1–3, 10]. The Western Trauma Association ET guidelines clearly suggest penetrating torso injury in patients with less than 15 min of prehospital CPR, as a primary indication to perform the procedure [2].

In 2006, Cothren and Moore reported an ET survival rate, following isolated cardiac injury, of 35% in patients presenting to ED in shock and 20% when vital signs were not present [10]. Tavares et al. in 1984 reported 56.8% of survival among “lifeless” or deteriorating patients with penetrating trauma, treated with an ET [13].

On the other hand, poor outcomes have been reported in ET following blunt trauma. While penetrating trauma is a clear indication for ET, ETs in blunt trauma is still matter of debate and many reviews have been published over the past years. Branney et al. suggested that ET after blunt trauma may be a relative indication in case of SoL at the initial paramedical evaluation [15]. The systematic review by Rhee et al. evaluated 24 studies and included 1024 blunt trauma patients, with a survival rate of 1.4% (15 patients). They concluded recommending ET for patients with blunt trauma with loss of SoL immediately before hospital admission or in the ED [16]. Similarly, the most recent case series reports a very low survival rate after ET performed for blunt trauma, especially in patients without signs of life at the time of arrival at ED, ranging between 0 and 15.8% [17, 18].

Unlike regions such as North America and South Africa where penetrating injuries predominate, the most common mechanism in the European setting is blunt trauma. The Scandinavian study by Pahle et al. included 263 ET performed in trauma patients, with a survival rate of 12% among the 82 blunt traumas. This was one of the highest survival rates of ET in blunt trauma; however, data seemed to be affected by some selection biases [7]. Most recently, Narvestad J.K. et al. (2016) published a systematic review about ET in European hospitals with an overall survival rate of 12.9% after blunt trauma [5].

To the best of our knowledge, this is the first Italian case series about ET. In our study, we have presented data on ETs performed at Maggiore Hospital in Bologna, one of the first Italian trauma centres which adopted the procedure in 1989. Between 1994 and 2009, ET was not performed after a critical review of 20 procedures with 100% mortality. Starting from January 2010, new literature on the topic about the utility of ET in patients in extremis or already in cardiac arrest after penetrating injuries, renewed interest for performing ET in our centre. ET survival rate of 5.8%, after the adoption of more liberal policy (supplementary figure 7) compared well with previous retrospective studies on ET that report survival rates ranging from 0 to 6%. In a recent review, Nevins and colleagues reported that the survival of ET, following blunt injury, was 5.2% and they conclude that ET for blunt trauma is now becoming more survivable and their paper has also demonstrated that survival following EDT is higher in the non-USA publications [19].The ETs performed at Trauma Center of Maggiore Hospital were done both in ED and in the OR. ET has been defined by Hunt P.A. et al. in 2006 as the procedure occurring either immediately at the site of injury, in the emergency department, or in the operating room, as an integral part of the initial resuscitation [20]. We concur with this definition and consider OR thoracotomies as ET as this can be considered part of the initial resuscitation. In addition, at our Institution, the OR is on the same floor as the ED and personnel are readily available 24/7. Fairfax et al. also recognize that ET is not confined to the ED and that if appropriate, the patient should be transferred to the OR for ET under more controlled conditions [21]. Kandler et al. reported different survival rates comparing ED (33%) and OR (83%) ETs; the literature supports favourable outcomes if thoracotomy is performed in the operating room compared to the emergency department; we believe that this is because these patients are likely to have less severe injury, have likely had time for optimum monitoring to be in place, and operating room conditions are usually better than that of the emergency department. The comparison between ET performed in ER and in OR should be a limitation of our experience but we consider the procedure as an integral part of the initial resuscitative procedure. These differences may probably be explained with the lower grade of injuries which allows transferring the patient to the OR [22].

The ET involves the utilization of expensive resources and this implies that if ET is not performed for a correct indication, a great economic waste is carried out. In 2007, a cost–utility analysis of ET demonstrated that ET is cost-effective when the procedure is performed for penetrating trauma following stricter inclusion criteria [23].

Since many of these patients are young and otherwise in good health, the topic of organ donation should be considered when SoL are absent or lost, before or after performing ETs. Schnuringer et al. presented 76 cases of blunt trauma patients who underwent ET where one patient survived (1.3%) and two donated their organs [24]. A recent study by Alarhayem AQ et al. concluded that clinicians should be more judicious in their decision to perform an ET, especially when SoL are lost upon arrival, and consider the potential for organ donation [25].

Recently, resuscitative endovascular balloon occlusion of the aorta (REBOA) has been proposed as an effective alternative for the exsanguinating patient in extremis due to non-compressible thoraco-abdominal injuries [26]. This is less invasive than ET and is considered as an effective bridge to surgery. Unfortunately, REBOA can only be used in specialized centres such as our institution as it requires specific training and equipment to insert the device. Nonetheless, ET may require a specific training and surgeons could improve the basic resuscitative manoeuvres by attending specific trauma course organized on the swine, such as in Italy.

Overall, ET is an uncommon procedure in European centres and this is evident in the limited number of papers published about the ET. Outcomes post-ET are also under-studied along with post-ET outcomes. Several Italian Trauma Centres have not published articles in recent years concerning ET. Thus, our paper would be a presentation of recent ETs performed at Maggiore Hospital in Bologna, during the past 6 years.

After critically reviewing our series and the European literature, we performed audits with trauma surgeons and trauma anaesthetist and we developed an algorithm (Figs. 1, 2) for indications for ET, to standardize the clinical management of patient in extremis or in cardiac arrest after trauma and try to improve the survival rate and outcomes and finally to optimize our hospital resources.

Regarding our experience at Maggiore Hospital, two main bias must be pointed out. First, the cohort of patients treated at our Institution is small and the statistical value of our results is limited. Second, we have collected data in a retrospective way; however, in the emergency traumatic setting, a randomized control trial for life-saving procedures would be considerably challenging.

## Conclusions

ET, performed both in the ED or in the OR, may be a heroic, life-saving procedure in selected patients. Penetrating trauma is an established indication for ET with good outcomes, especially when a single cardiac injury is detected and repaired, relieving a cardiac tamponade. ET in blunt trauma is a debated indication since it has poor outcomes (survival 0–15.8%) and limited cost–benefits. Nowadays, few reports from European trauma centres are available about ET and only one systematic review has been published in 2016, with favourable overall reported outcome, also after blunt trauma. Performing ET for the right indication may be cost-effective, especially when the patient does not develop neurological sequelae; however, when neurological impairment affects the patient or the health of the care providers is exposed to possible blood-borne infections, the procedure may be a waste of resources. A different potential outcome, which can be pursued with the enlargement of ET indications, is organ donation. More investigations are needed to encourage the trauma teams to perform ET more frequently, especially young and otherwise healthy trauma patients without SoL in ER, with the goal to increase organ donation rates.

## Electronic supplementary material

Below is the link to the electronic supplementary material.

Video #1: EDT Clamshell in a patient in CC after blunt trauma with massive destruction of the R lung. R pulmonary hilum clamping. https://youtu.be/CCCt17Ig9No (mp4 86683 kb)

Video #2: EDT Clamshell in a patient in CC with Cardiac Injury. Cardiac Repair. https://youtu.be/3X2qUTub_ss (mp4 37709 kb)

During the ET, many manoeuvres should be performed, such as opening the pericardium or aortic cross-clamping (JPEG 3385 kb)

Twisting of the pulmonary hilum for controlling massive hilar injuries (JPEG 2510 kb)

A clamshell incision is performed at 4°–5° intercostal space, below nipple in males and in the infra-mammary crease in females (JPEG 2084 kb)

Clamshell thoracotomy provides a better exposition of the thoracicorgans and vessels (JPEG 2591 kb)

Outcome before discharge on POD 30 (JPEG 245 kb)
