# Supplementary figures and images for: Outcomes and indications for emergency thoracotomy after adoption of a more liberal policy in a western European level 1 trauma centre: 8-year experience
Source: Updates Surg. 2018 Dec 26;71(1):121–7. doi: 10.1007/s13304-018-0607-4 (PMC6450838; doi:10.1007/s13304-018-0607-4)

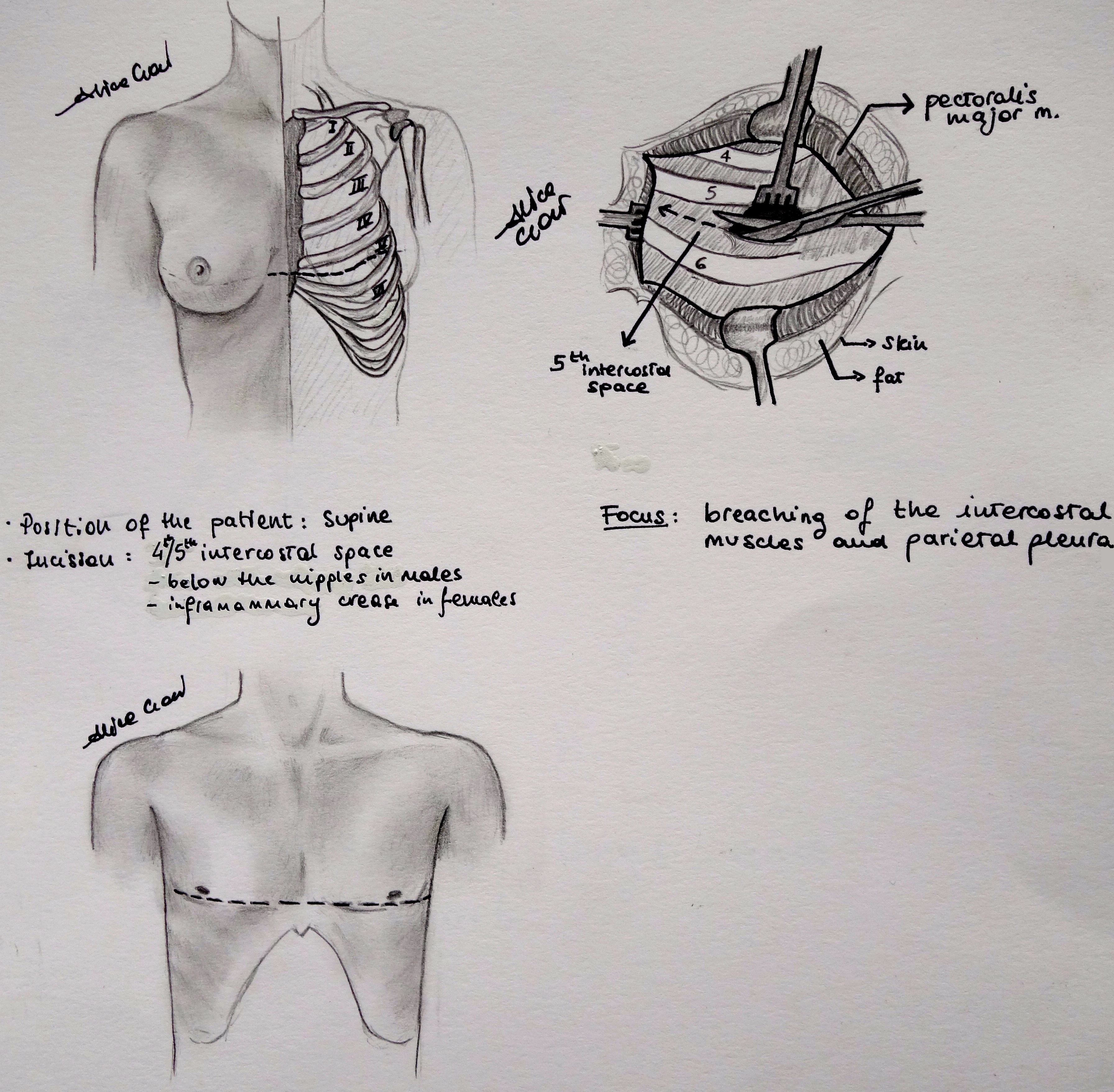

Supplement: Supplementary file 3 — During the ET, many manoeuvres should be performed, such as opening the pericardium or aortic cross-clamping (JPEG 3385 kb) [file 13304_2018_607_MOESM3_ESM.jpg]

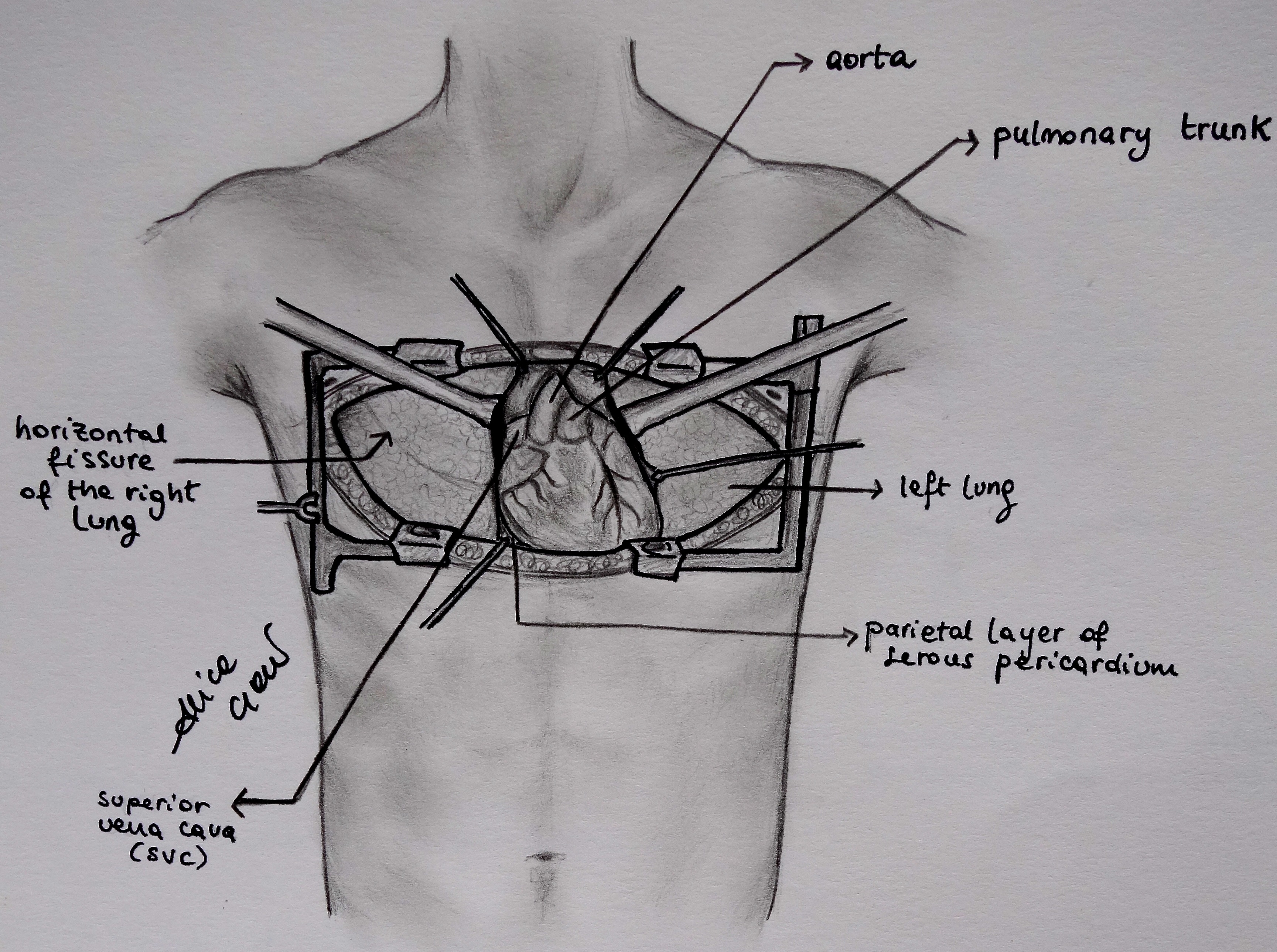

Supplement: Supplementary file 4 — Twisting of the pulmonary hilum for controlling massive hilar injuries (JPEG 2510 kb) [file 13304_2018_607_MOESM4_ESM.jpg]

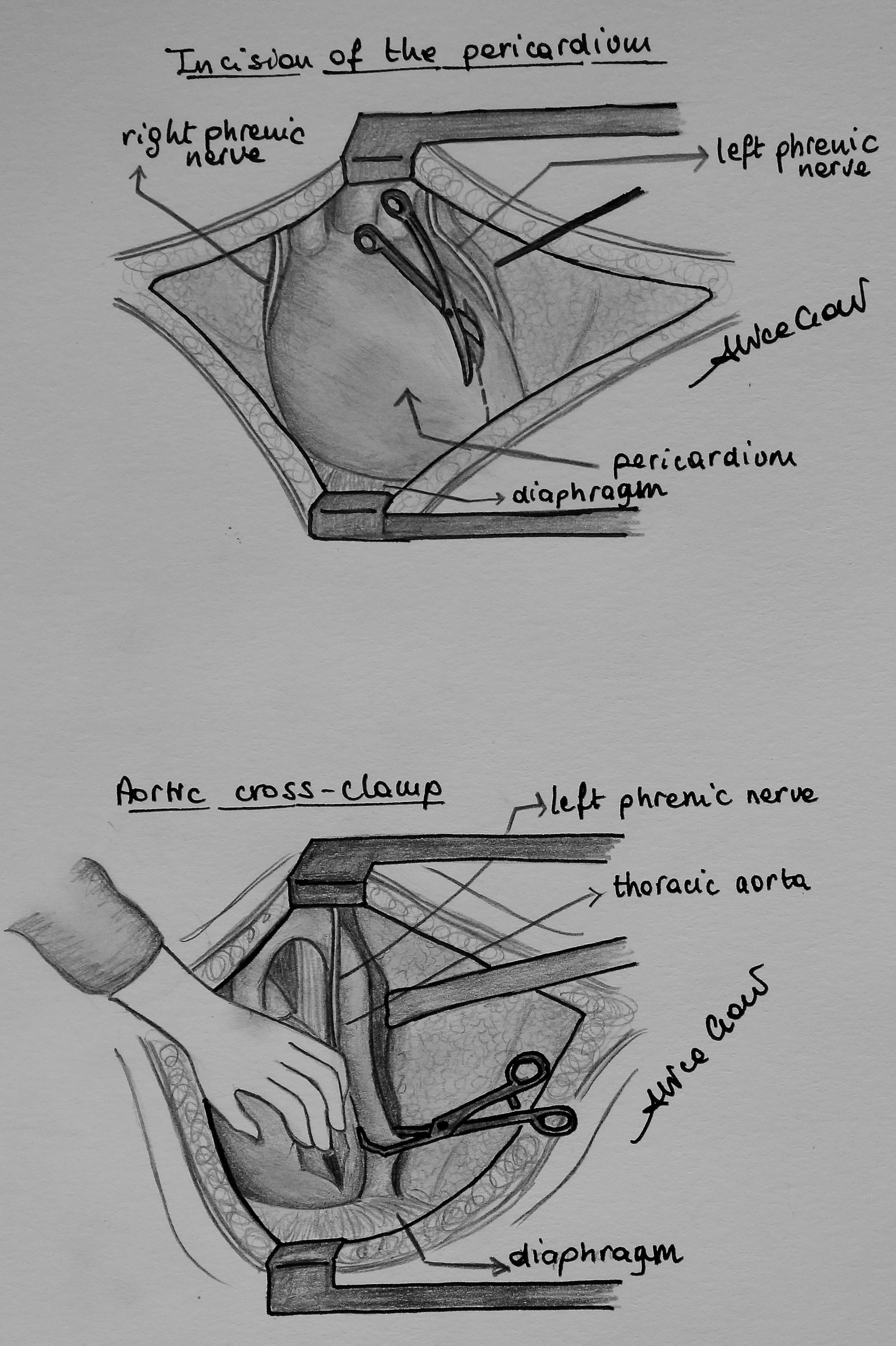

Supplement: Supplementary file 5 — A clamshell incision is performed at 4°–5° intercostal space, below nipple in males and in the infra-mammary crease in females (JPEG 2084 kb) [file 13304_2018_607_MOESM5_ESM.jpg]

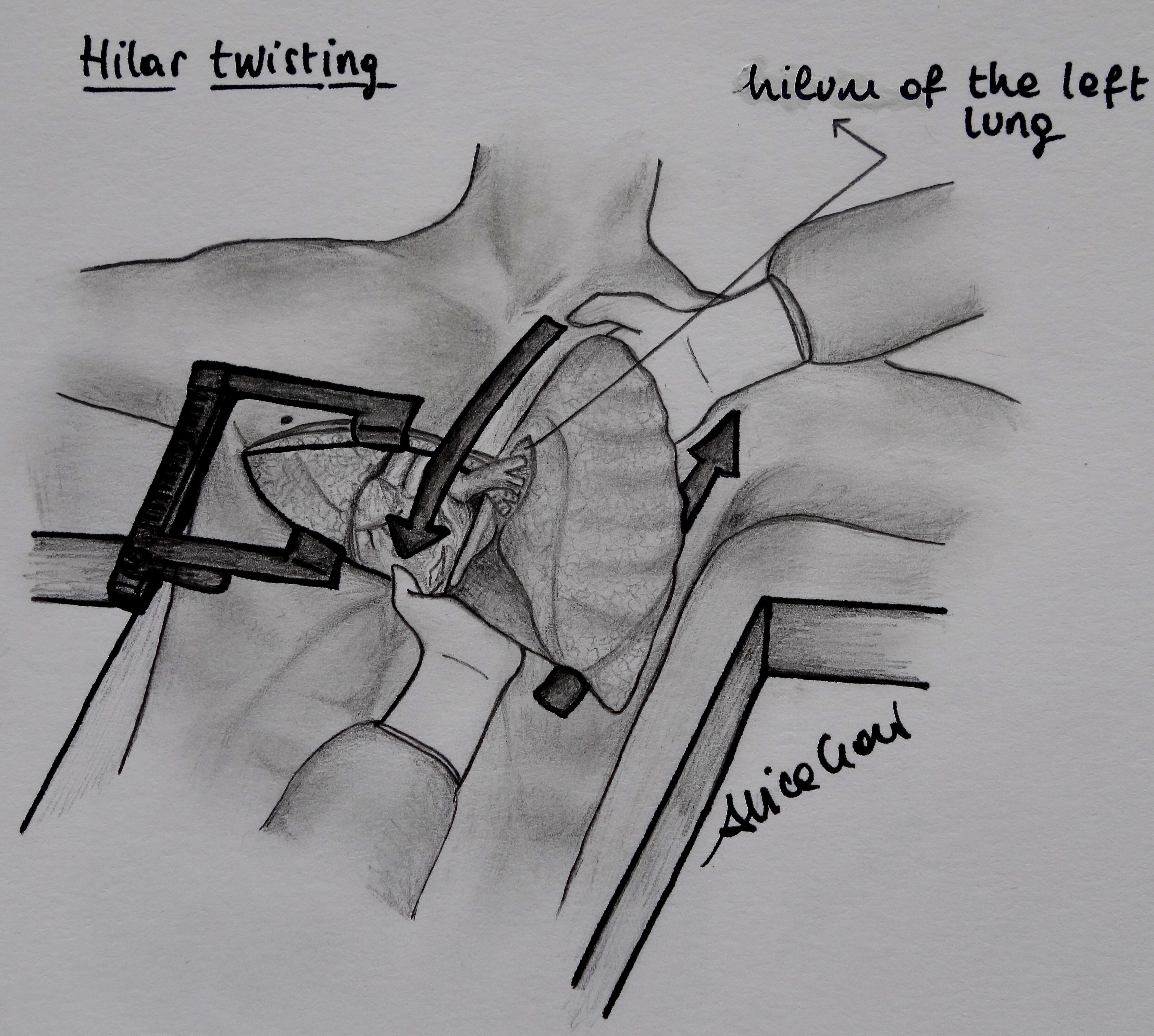

Supplement: Supplementary file 6 — Clamshell thoracotomy provides a better exposition of the thoracicorgans and vessels (JPEG 2591 kb) [file 13304_2018_607_MOESM6_ESM.jpg]

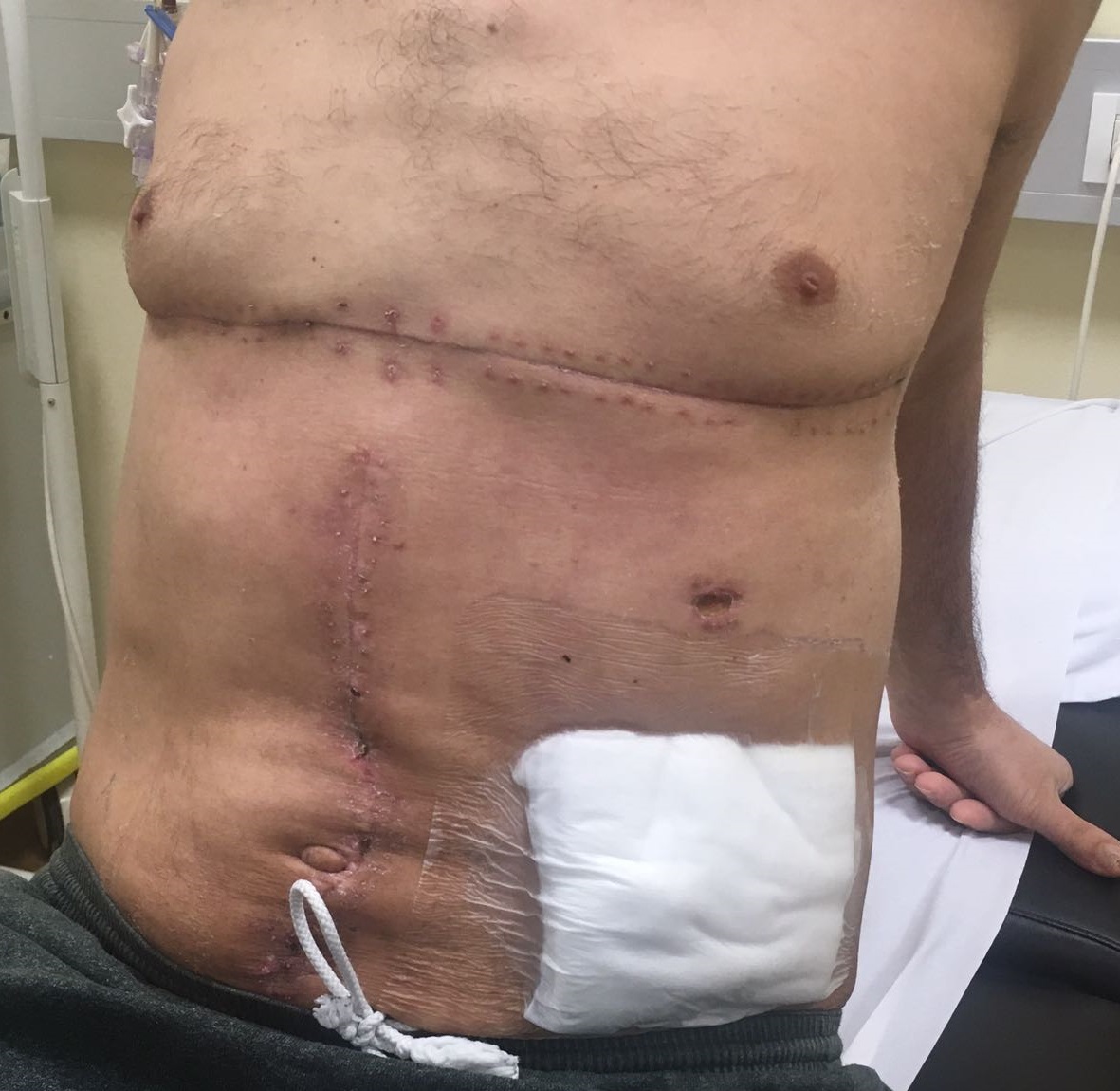

Supplement: Supplementary file 7 — Outcome before discharge on POD 30 (JPEG 245 kb) [file 13304_2018_607_MOESM7_ESM.jpg]
